# Peer Review for “Checklist Approach to Developing and Implementing AI in Clinical Settings: Instrument Development Study”

**DOI:** 10.2196/69870

**Published:** 2025-02-20

**Authors:** Anonymous

**Keywords:** artificial intelligence, machine learning, algorithm, model, analytics, AI deployment, human-AI interaction, AI integration, checklist, clinical workflow, clinical setting, literature review

*This is the peer-review report for “Checklist Approach to Developing and Implementing AI in Clinical Settings: Instrument Development Study.”*

## Round 1 Review

###  General Comments

This paper [1] construct a checklist to support the development and implementation of artificial intelligence (AI) in clinical settings. I only have some minor comments.

### Minor Comments

1. Comparison with existing checklists: Please add a comparison with some of the existing checklists.

2. Inconsistency in the number of studies: The authors initially stated that they included 20 studies, but later mentioned 23. Please clarify.

3. Appendix visibility: The appendix is not visible.

4. Abbreviation consistency: The abbreviation “IQR” appears multiple times. Please ensure it is clearly defined and used consistently.

## Abbreviations

AI: artificial intelligence
